# Brave New Worldview

**DOI:** 10.1007/s11948-014-9553-9

**Published:** 2014-05-16

**Authors:** Taft H. Broome

**Affiliations:** 11 Parkside Road, Silver Spring, MD 20910 USA

John Kenneth Galbraith once let slip that a “mild revulsion” to students had him contemplating retirement from Harvard. So, in 1973, he opted to do a television series:

We settled on the title “The Age of Uncertainty” for the series. It sounded well; it did not confine thought; and it suggested the basic theme: we would contrast the great certainties in economic thought in the last century with the great uncertainty with which problems are faced in our time. In the last century capitalists were certain of the success of capitalism, socialists of socialism, imperialists of colonialism, and the ruling classes knew they were meant to rule. Little of this certainty now survives. Given the dismaying complexity of the problems mankind now faces, it would surely be odd if it did. (Galbraith 1977, p. 7)

For Galbraith’s part, his experience as an advisor to US President John F. Kennedy revealed a loss in the certitude with which intellectuals once influenced the world’s leaders. For him, the causal mechanism of that loss was the complexity of mankind’s problems.

In the first half of the twentieth century, Alfred North Whitehead contributed much to the challenge faced by Harvard University of fitting the practical education of its school of business into a culture dominated at that time by liberal education. He said,

There is a curious illusion that a more complete culture was possible when there was less to know. Surely the only gain was that it was more possible to remain unconscious of ignorance. It cannot have been a gain to Plato to have read neither Shakespeare, nor Newton, nor Darwin. The achievements of a liberal education have in recent times not been worsened. The change is that its pretensions have been found out. (Whitehead 1929, p. 57)

For Whitehead’s part, learning enlarges as problems become more complex, and the certitudes of experts are regulated less by complexity than cultural completeness, i.e., a harmony, proper for a culture, of liberal or intellectual education and practical education.

For my part, Whitehead was right: complexity fails as a probable cause of uncertainty among experts; incomplete cultures seem the more likely cause. Yet, Galbraith’s Age of Uncertainty, last of the Globalizing Ages covering the spread of humankind and/or human cultures over planet Earth, leaves the transition to a Globalized or Global Age to the twenty-first century. Can editorial boards of scholarly journals better ready the academy for the Global Age? Can the *Science and Engineering Ethics* (SEE) editorial board uniquely ready the academy for the Global Age?

The paradigm of the experts in the Globalizing Ages of Modern Western Civilizations was a shared worldview that divides experts into intellectual and practical categories; legacies, respectively, of Athens and Rome. Table 1 organizes some terms I shall use later. I shall herein contrast empirical reasoning with theoretical reasoning and let it suffice to say that praxis consists in ideas for making and executing plans of action, that practice consists in praxis-informed psycho-motor actions, and that the pure/applied dichotomy references any attempt to discriminate the general and the specific.^1^

**Table 1:**
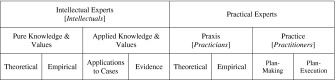
Paradigm analysis variables

Whitehead’s vision of a complete culture or proper harmony of intellectual and practical education puts light on persistent struggles in Western civilization between intellectual and practical experts, particularly in science and religion. Perhaps most anti-intellectuals are not experts. Amateurs comprise an intermediate case. Pseudo-intellectuals comprise a different sort.

In 1867, Karl Marx (2000) planted the seeds of a worker’s revolution in the Industrial Revolution. In Marx’s factory, workers unionize to protect themselves from exploitation by capitalists and from oppression by intellectuals and practicians. All personnel function along lines of responsibility, authority and accountability that mimic military functioning. Metaphorically: capitalists are general officers, intellectuals and practicians are officers, skilled workers are sergeants, and laborers are soldiers; but military officers oppress neither sergeants nor soldiers.

Extrapolated into the Age of Uncertainty, Marx’s island of estranged and alienated workers is structured but does not function as the mainland. A master mechanic may play a role on the island that was designed on the mainland to function there as an officer, but if he plays intellectual on this unified island he will risk being called an oppressor by his fellow islanders. If he opts to function in this role as a sergeant, he is a pseudo-intellectual. For mainlanders, Marx’s island is an upside down world—and vice versa.

The extrapolation continues. Whereas intellectuals infer conclusions individually, pseudo-intellectuals decide them collectively. Whereas intellectuals assess conclusions with qualitative and quantitative evidence, pseudo-intellectuals assess them by weight of the evidence. Whereas intellectuals work hard, pseudo-intellectuals obey the gospel of hard work. As scarcity of jobs in the factory drive islanders to the mainland, pseudo-intellectuals clash with intellectuals. Consequently, the general education and its core curricula in secondary and higher education on the mainland would spread the island worldview among such mainlanders as may be susceptible to it. In fact, the paradigm of the experts in the Age of Uncertainty is the worldview of the mainland punctuated by episodes of pseudo-intellectualism.

Husserl argues that if human consciousness is merely material, a part of physical nature, it can never be a foundation for rational certainty. Naturalism has brought upon us, says Husserl, the present crisis of the loss of a belief in any absolute certainty, any rational truth. And Husserl makes it clear that this is not only an intellectual crisis of the lack of any certainty at the foundation of our thought, but a social and political crisis as well: if, for European man, no belief has certainty, then European man has no truth to be his shield against the rise of fascism and its appeal to irrationalism. (Levine 1984, p. 395)

In the early Age of Uncertainty (1900–1960s), the American Association of University Professors (AAUP) was founded (1915) to protect academic freedom. Pseudo-intellectuals demanded conformity and Jean Paul Sartre, though refusing the Nobel Prize in 1964, and Nobel Laureate T. S. Eliot used existentialism to counter the union impulse of conformity. Pseudo-intellectuals damned philosophy and Ludwig Wittgenstein strengthened the language of intellectuals. Positivists and pragmatists celebrated empirical science. Agatha Christie’s lovable intellectual, Poirot, out-smarted her insufferable pseudo-intellectuals (Christie 1984). In 1920, a pamphlet on communism was found in a Howard University (HU) library. The university’s President Mordecai Wyatt Johnson, in spite of pressure from one Congressional committee (1933) and under threats to the university’s Congressional appropriation from another Congressional committee (1934), kept the pamphlet in HU’s library (Dyson 1941). McCarthyism came 20 years later and Richard Hofstadter won the Pulitzer Prize for *Anti*-*Intellectualism in American Life* (1963).

In the late Age of Uncertainty (1970 to today), the intellectual heroes are the television program NOVA; the History Channel; the Nobel Prize winners Joseph Rotblat and Pugwash Conferences in 1995 and, in 2007, the Intergovernmental Panel on Climate Change (IPCC) and US Vice President Al Gore; and other thoughtful and thought-provoking, paradigm-shifting works. In 2006, Pulitzer Prize winning author Chris Hedges published *American Fascists: The Christian Right and the War on America* (2006). The attempts of intellectuals to influence political and business leaders were split and well-justified. The attempts of pseudo-intellectuals were unanimous and poorly justified. Such clashes were not dialectics promising syntheses of the two sides, but zero-sum games with high stakes.

A projected paradigm of the experts in the Global Age is the shared normal worldview of the Age of Uncertainty shifted towards practicians; pseudo-intellectuals are lepers, and China is #1. A call of the Global Age is to protect societies (Hill 2014) so that they will not merely survive but indeed flourish amid global economic meltdowns, global warming, globally virulent viruses and cybercrime; amid the growth, diversification and stratification of the world’s population; and amid new Godzillas from the collective unconscious symbolizing the mega-asteroids, mega-volcanoes and mega-tsunamis foreseen in the now imminent onset of global climate change.

Today, the transition to a Globalized or Global Age suggests that deep introspections by editorial boards into their scholarly literatures would indicate whether the academy has drifted too far from ideas or focused too narrowly on facts to answer the call of the Global Age. Table 1 is a map for such introspection.

That our universities have grave shortcomings for the intellectual life of this nation is by now a commonplace. The chief source of their inadequacy is probably the curse of departmentalization. (Frankfurter 1948, p. 24)

Today, *Science and Engineering Ethics* is uniquely positioned to answer the call of the Global Age for a body of learning whose intellectual growth is proportionate to its practical growth. Spanning the full range of the analysis variables in Table 1, SEE can uniquely cultivate a paradigm shift: not to practicians, but to Whitehead’s complete culture, a proper harmony of intellectuals, practicians and practitioners. Does this harmony have a sign? There is the sign post up ahead: *Brave New Worldview*.

## Footnotes

1 The terminology laid down here was introduced in Broome (2011).
